# The Paradoxical Role of Perceived Control in Late Life Health Behavior

**DOI:** 10.1371/journal.pone.0148921

**Published:** 2016-03-14

**Authors:** Judith G. Chipperfield, Raymond P. Perry, Reinhard Pekrun, Petra Barchfeld, Frieder R. Lang, Jeremy M. Hamm

**Affiliations:** 1 Department of Psychology, University of Manitoba, Winnipeg, Canada; 2 Department of Psychology, University of Munich, Munich, Germany; 3 Institute of Psychogerontology, University of Erlangen-Nuremberg, Nuremberg, Germany; Federal University of Rio de Janeiro, BRAZIL

## Abstract

Research has established the health benefits of psychological factors, including the way individuals appraise outcomes. Although many studies confirm that appraising outcomes as controllable is adaptive for health, a paradoxical possibility is largely ignored: Perceived control may be detrimental under some conditions. Our premise was that appraising health as controllable but at the same time ascribing little value to it might signal a dysfunctional psychological mindset that fosters a mistaken sense of invincibility. During face-to-face interviews with a representative sample of older adults (age range = 72–99), we identified individuals with such a potentially maladaptive *“invincible”* mindset (high perceived control and low health value) and compared them to their counterparts on several outcomes. The findings were consistent with our hypotheses. The *invincibles* denied future risks, they lacked the activating emotion of fear, and they visited their physicians less often over a subsequent five-year period. Moreover, in contrast to their counterparts, the *invincibles* did not appear strategic in their approach to seeking care: Even poor health did not prompt them to seek the counsel of a physician. The recognition that psychological appraisals are modifiable highlights the promise of remedial methods to alter maladaptive mindsets, potentially improving quality of life.

## Introduction

Decades ago researchers began acknowledging the critical role of psychology in the etiology of disease and the promotion of good health [1]. The impact of psychological factors on health is clearly illustrated by the placebo effect wherein benefits accrue from a mere expectation that a drug will be effective. In a similar way, an expectation that health is controllable can generate positive consequences. This expectation of influence over outcomes, referred to as *perceived control (PC)*, has been portrayed as a “hallmark of American culture” [2].

PC varies by domain (e.g., work, finances, relationships) and is lower in some domains for older relative to younger adults [3]. This underscores the need for a domain-specific approach when studying PC and highlights the importance of evaluating its role in late life when the capacity to influence outcomes is threatened by many challenges. The present study assessed psychological appraisals of control (influence) in the domain of health among older individuals, some of whom were nearly centenarians. Our approach departs from the majority of past studies that examine control as an isolated appraisal. Instead, appraisals of control were considered within a broader motivational framework that included the appraised value of health.

### Previous Research: Consequences of Perceived Control (PC)

An extensive literature on PC shows it has relevance for a vast array of outcomes, including physiological indicators and brain activation. For example, positron emission tomography (PET) and functional magnetic resonance imaging (fMRI) studies show that PC triggers neuronal brain activation, facilitates regional brain metabolism, and plays a role in sensory discriminative aspects of how pain is processed [4–7]. Results of experiments show that believing a stressor can be modified is accompanied by a reduced cortisol response [8], and field research in natural settings indicates that PC is linked to physiological processes and systems (e.g., cytokine secretion, metabolic biomarkers) that are critical for the functioning of the pulmonary and cardiovascular systems [9–10]. Further evidence suggests the benefits of PC extend to general health and even to survival [10–19].

### Moderators that Qualify the Effects of PC

Despite overwhelming support for an adaptive role of PC, positive consequences do not always follow from appraising an outcome as controllable. The PC effect is shown to be qualified or moderated by other factors, including age and functional deficits. For example, PC appeared to be protective with regard to self-rated health in one study, but only for the oldest old who had functional limitations [19]. In another study, PC was beneficial in predicting less use of health services (laboratory tests, hospitalizations, and hospital stays), but only for those who were restricted by their arthritis [20].

A critical moderator of PC is implied in *expectancy-value theories* (EVT) that date back to the 18^th^ century [21]. EVT theories are commonly used to explain human motivation, [22–25] positing that motivated behavior occurs only when an expected outcome is also sufficiently valued. New variants of EVT theory continue to emerge, such as the *control-value theory* [26–27], which is particularly relevant for our purposes because expectations are embedded in one’s appraised capacity to influence outcomes. According to the theory, appraising an outcome as controllable is most beneficial when the outcome is also valued. Expecting to have control over an outcome that is regarded as trivial (devalued) may be of little consequence, since such a mindset would presumably fail to cultivate activating emotions and engagement.

Several studies on achievement in everyday life and those conducted in the social and health domains provide support for this premise that the effect of PC is qualified by value [16, 28–32]. Of greatest relevance to our research are the studies that directly assess a PC x health value (HV) interaction on outcomes such as exercise, diet, smoking, and seeking of health information. For example, in one study, a beneficial effect of PC (internal locus) on information seeking emerged only for those who valued their health [32].

By focusing on whether high HV magnifies the benefits of PC or whether low HV limits those benefits, researchers have largely ignored another possibility. Although seemingly paradoxical, PC may be detrimental when health is devalued. For example, the reckless behavior of an alcoholic who cares little about health may be exacerbated by a belief that he has the will power to simply stop drinking when he chooses. Some support for a detrimental effect of PC has arisen in studies of older adults’ emotional well-being [33]. When high PC does not match with the expectation of positive outcomes, subjective well-being appears to suffer [34]. Detrimental effects of PC would also be compatible with harmful consequences of related constructs like unrealistic optimism [35] and illusionary positive views of the future such as when one unrealistically overestimates future satisfaction [36]. Thus, there may be conditions under which PC becomes maladaptive.

A detrimental role of PC seems especially likely if an expectation that health can be controlled coexists with a devaluing of health. This combination of appraisals might foster a misguided and mistaken sense of invincibility, resulting in procrastination that curbs behaviors like going to the doctor [37]. For example, an older woman who notices swelling in her legs is not likely to seek health care if she devalues her health (low HV) and believes she can alleviate the swelling by elevating her legs (high PC). Such an invincible mindset could foster disengagement with the health care system that is thought to result in missing timely diagnoses and treatments [38–41]. If this way of thinking becomes more entrenched in later life, it could be increasingly more dysfunctional, especially if it operates at times when care is most needed.

Disengagement and self-neglect appear to be exacerbated by other cognitions and emotions that may accompany an invincible mindset. For example, individuals who appraise their health as controllable and devalue it may also feel fearless and impervious to risks. A classic example would be the fearless, risk-taking teenager who has exaggerated feelings of power (high PC) and who devalues or ignores health and safety (low HV). Although an older adult is not likely to express the reckless behavior of the teenager, such a mindset may foster maladaptive behaviors involving disengagement. The importance of such cognitions and emotions are highlighted by research suggesting that some fear motivates help seeking [42–43] and that attending to future risk prompts proactive health behaviors (e.g., obtaining vaccinations) [44]. Thus, in the context of seeking health care, it may be toxic to possess a mindset characterized by an absence of fear, a failure to acknowledge risk, and a confluence of appraisals (high PC-low HV).

### Dual Appraisals of Control and Value in a Health Context

The major objectives of the present study were to consider whether an invincible mindset (high PC-low HV) is characterized by other potentially maladaptive cognitive and emotional characteristics and to assess whether this mindset has potential long-term consequences for seeking health care. Our study has several advantages over past research that has assessed the dual appraisals of control and value in a health context. *First*, extending past approaches that emphasize the positive consequences of PC [16–20, 45], we tested a novel premise that PC is potentially maladaptive when combined with a low value appraisal. *Second*, to analyze the potential consequences of these appraisals, we draw on a representative sample of older adults, departing from most existing studies that have employed convenience samples of young adults [30–31]. *Third*, by examining objectively assessed physician visits, we extend the narrow range of previously studied outcomes (e.g., smoking) that have primarily relied on self-report [30]. *Finally*, our analysis of physician visits involved a lengthy follow-up period (five years).

**Fig 1:**
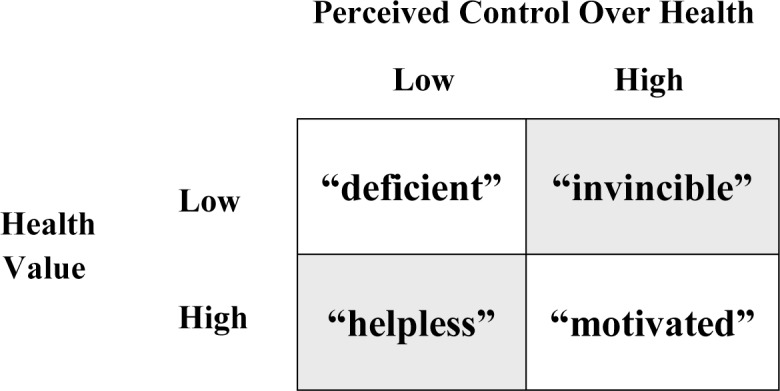
Mindset Matrix: Groups with Varying Appraisals of Perceived Control and Health Value.

For the present study, individuals were classified into a 2 x 2 appraisal group or *mindset matrix* based upon their joint control and value appraisals (Fig 1). Combinations involving either a high score on both appraisals of PC and HV or a low score on both (unshaded cells) can be distinguished from combinations involving a high score on one appraisal and a low score on the other (shaded cells). Provisional labels were selected to represent mindsets in each cell of the matrix. These intuitively meaningful labels are adopted as simple conceptual heuristics and are not intended to portray unchangeable personality types. The *invincible* label represents those who believe they can control their health but ascribe low value to it (high PC-low HV), a mindset that presumably corresponds to feeling imperviousness to harm. The *motivated* label identifies individuals who believe they can control their health and highly value it (high PC-high HV); *deficient* individuals are those who neither appraise their health as controllable nor value it (low PC-low HV); and *helpless* individuals are those who believe their health is uncontrollable, but ascribe high value to it (low PC-high HV).

To examine the possibility that the invincible mindset (high PC-low HV) is also characterized by other potentially dysfunctional psychological characteristics, it was predicted that, compared to other appraisal groups, the *invincibles* would express greater denial-of-risk (H1) and be more likely to report an absence-of-fear (H2). Physician visits were examined to consider whether an invincible mindset increases the risk of disengaging from health care. The *invincibles* were expected to visit their physicians less frequently than their counterparts in the other appraisal groups (H3), even after statistically ruling out the possibility that underutilization of health services could be due to them being healthier and having less need for care.

A final analysis directly examined the role of health status in physician visits for the four appraisal groups. We reasoned that it is when people are in poor health that it is most strategic to seek care. Thus, we predicted that those in poor health would visit their physicians more often than their counterparts; however, we did not expect the *invincibles* to seek the counsel of a physician more so when in poor health (H4).

As outlined below, support was found for each of these hypotheses. The *invincibles*’ were shown to deny risks and to be fearless, suggesting a toxic mindset. They also visited their physicians less frequently, and appeared less strategic in their approach to seeking care.

## Materials and Methods

Our study was comprised of a subset of community-dwelling individuals who completed in-home interviews as part of the 35-year (1971–2006) Aging in Manitoba (AIM) Project. Relevant data for the variables in the present study are available in supporting information files (see S1 File and S2 File). This research was approved by Manitoba Health’s *Health Information Privacy Committee* and by the University of Manitoba’s *Health Research Ethics Board*. All participants provided their written consent to participate in interviews and to have the interview data linked to their health records. The variables are described following an overview of the database and participants.

Among the strengths of the AIM Project are rigorous tracking strategies that produce outstanding retention, as well as intensive sampling and recruitment procedures that result in high response rates and representative samples. The AIM sample is comparable to the overall provincial population [46]. AIM interview data are merged with other sources that permit the linkage to objective measures (e.g., physician visits) and to data from a satellite study, the Successful Aging Study (SAS) that was initiated in 1996.

In the SAS satellite study (*n* = 353), participants were interviewed to obtain focused information on psychological appraisals, emotions, and other cognitions. For the present purposes, participants were excluded (*n* = 115) if they did not have a valid value on the appraisal group variable that was created by combining appraisals of control and value (see variables). The maximum potential sample size for our analyses was 238, although sample sizes differed across hypotheses for reasons that are clarified below.

### Variables

#### Baseline (1996) Variables

Descriptive statistics are based on the maximum number of potential individuals retained for analyses (*n* = 238). Demographic variables included: age (*M* = 79.96, *SD* = 5.85, *range* = 27.00), gender (1 = *female*, *n* = 150, 63.0%; 2 = *male*, *n* = 88, 37.0%) and monthly income acquired from all sources (e.g., pensions/allowances such as Old Age Security, private pensions, wages, dividend interest) in Canadian dollars (*M* = $1,464.83, *SD* = 1,103.18, *range* = 5453.00).

A three-step procedure was used to create a physical health status index [47]. *First*, participants reported the occurrence (yes, no) of 22 chronic health problems encountered within the previous year (e.g., arthritis, palsy, emphysema, cancer, stroke and heart-related problems, etc.). *Second*, an objective estimate of the severity of each condition was acquired by borrowing severity scores from the revised seriousness of illness rating (SIRS-R) scale [48–49], or by having medical residents rate the seriousness of conditions that were not included in the SIR-R. *Third*, for each participant, a mean was calculated over the seriousness scores for each reported condition such that higher scores corresponded to poorer health (*M* = 304.66, *SD* = 194.87). Because the severity of chronic conditions score takes into account objectively-determined seriousness of conditions, it is more sensitive than a self-report of chronic conditions that is often used [50]. In addition to this continuous health status variable, a dichotomous variable was created, allowing for a comparison of those in poor (*M* = 182.62, *SD* = 107.59) versus good (*M* = 120.83, *SD* = 76.30) health. The use of a dropped median-split procedure resulted in the loss of one case.

Functional status was assessed using a classic approach [51] that determines a participant’s reported ability to undertake basic activities of daily living (BADL) and instrumental activities of daily living (IADL). BADL items (*n* = 11) that are central to daily functioning included getting about the house, getting in and out of bed, washing, bathing, grooming, dressing, eating, and so on. IADL items (*n* = 12) that allow one to live independently included doing housework, preparing a hot meal, shopping, managing finances, reading, and so on. We calculated a mean over activities a participant could perform without any help (0 = *help is required*, 1 = *help is not required*) so that a higher score reflected better functional status (*M* = 9.93, *SD* = 1.44, *α* = .82, *range* = 3.0–11.5).

#### Appraisals of Control and Value

The appraisal of control was assessed using a common domain-specific approach [3] to estimate PC by asking participants about the extent to which they could personally influence their health (1 = *almost no influence*, 10 = *total influence; M* = 7.34, *SD* = 2.34, *range* = 9.00). Since the conceptual integrity of our predictions required a domain-specific measure of PC, this direct estimate of control in the health domain was preferred over a composite scale combining control appraisals across various domains. A judgment of influence over health has high face validity, and existing evidence shows that single-item quantitative appraisals can have strong predictive validity [18, 52].

The appraised value of health was determined by participants’ agreement (1 = *strongly disagree*, 7 = *strongly agree*) with six statements, four that were taken from an existing Health Value (HV) scale (“If you don’t have your health you don’t have anything;” “There are many things I care about more than my health;” “Good health is of only minor importance in a happy life;” “There is nothing more important than good health” [53]. Where necessary, item coding was reversed so that higher scores corresponded to greater health value.

This scale has been used with populations ranging in age from 18 to 93 years [31, 54–55]. Prior studies [30–31] support its psychometric properties by demonstrating test-retest reliability (*r* = .86 over 18 months); predictive validity (HV significantly predicts health behaviors); and internal consistency (*α* range = .63 to .71). In our study, two items were added to further improve the internal consistency: “It is important to do things that will reduce the likelihood of bad health;” “It is important to do things that will increase the chances of good health.”

The alpha for our six-item HV index was .72, all of the inter-item correlations were significant, and a principal component factor analysis showed that the items were represented by a single factor (eigenvalue = 2.53, explained variance = 42.1%) with high item factor loadings (range = .61 to .69). Notably, the distributions for the HV items showed inter-individual variability, with some individuals scoring in the low or mid-range. For example, nearly one third of participants agreed with the statement “There are many things I care about more than my health.” This contradicts the assumption that health is universally valued and is compatible with anecdotal accounts of individuals who value other outcomes over health (e.g., success or knowledge). The scores for the six items were transformed (reflected square root transformation) and averaged to create a continuous HV index (*M* = 1.75, *SD* = .51, range = 0.66–2.45). A high HV score captures strong agreement that health is valued.

A four-level appraisal group variable was created to map onto the mindset matrix (see Fig 1). The use of a dropped median-split method to identify high and low cut-off points for the appraisals of control and value ensured a meaningful separation between appraisal groups while still retaining an adequate sample size (*n* = 238) for analysis [56]. The majority of participants were classified as deficient (*n* = 73, 31%) due to neither appraising their health as controllable nor valuing it (both PC and HV were below the median). The next largest was the motivated group (*n* = 65, 27%) that would typically be considered the most advantaged, followed by the helpless group (*n* = 59, 25%) that would be regarded as vulnerable, and the invincible group (*n* = 41, 17%) that we propose is the most disadvantaged.

#### Denial-of-Risk

Denial-of-risk was assessed among individuals who were exposed to a threat-provoking experimental task. Respondents who had completed a lengthy SAS interview were precluded from participating in this task due to concerns about the additional burden that would be imposed by the task. Among the precluded individuals were those who had experienced a prior heart attack. Thus, these participants were spared from the stress of recalling their real-life heart attack.

The experimental task was administered to the subset of SAS participants who had responded to a short interview. The task required them to imagine being a patient hospitalized for a health crisis (heart attack) and being told by a physician “…you have several blockages.” Threat was induced by a message about the risk of a future heart attack (low, unknown, or high). Given the usual challenges to the validity of experimental manipulations, we took several steps to enhance the legitimacy of the message: (a) the hypothetical threat was delivered by a credible source (a physician); (b) the message was believable (doctors often impart warning messages to motivate healthy behavior in their patients); and (c) the threat (heart attack) was one that is familiar to older adults. Many older adults contemplate the possibility of a future heart attack, even seeking emergent care for a suspected heart attack.

Denial-of-risk was assessed following the threat induction. The denial-of-risk score was derived from participants’ estimates of their *own* chance of having another heart attack (0 = *no chance*, 100 = *definite*; *M* = 54.58, *SD* = 24.93, *range* = 100.00), as well as *another* person’s chance (0 = *no one*, 100% = *every person*; *M* = 62.11, *SD* = 20.56, *range* = 88.00). A difference score was calculated for those who had provided both estimates of self and another by subtracting one’s own estimated risk from the estimate of another’s risk (other’s risk–own risk). A positive score that reflected seeing less risk for the self than another was interpreted as more denial-of-risk.

From among the potential sample, participants were excluded if they had not received the experimental task or had been assigned to the low-threat condition that we assumed would prime them to believe there was nothing to deny. Support was found for the assumption that the low threat condition would not activate denial-of-risk by showing that the *invincibles* exposed to the low-threat condition expressed very little denial-of-risk (*M* = 5.0) relative to their counterparts who were exposed to the unknown- (20.0) or high- (18.8) threat condition. For the subset of participants who had valid scores (*n* = 64), the mean denial-of-risk score was 7.53 (*SD* = 18.35, range = -30 to 80).

#### Absence-of-Fear

Absence-of-fear was assessed by asking potential participants about the extent to which they experienced fear in the past two days (0 = *never*, 1 = *once in a while*, 2 = *fairly often*, 3 = *very often*). Five cases were excluded due to missing values (*n* = 233). This continuous score that had a highly skewed distribution was recoded into absence-of-fear (84%) versus presence-of-fear (16%). As outlined elsewhere, the short two-day time frame was intended to enhance reliability because, despite having a well-preserved memory for emotional information, proximal emotions are more reliably assessed than are distant ones [57–58]. For example, the recall of fear experienced yesterday is apt to be more accurate and less biased than is the recall of fear that was experienced months ago [59–60].

#### Ambulatory Physician Visits

Objective data on ambulatory physician visits were obtained from the Manitoba Health Registry, a provincial administrative database. Patient contacts with the universal health care system are recorded by physicians in order to secure pay-for-service. Thus, there is a high motivation to report all contacts, resulting in reliable and comprehensive population-based data on physician visits.

Our analyses focused on the numbers of physician visits that occurred in five years following the 1996 SAS interview. *Cumulative counts* were generated, capturing the number of visits occurring between the interview date and the end of each follow-up period. The counts were generated after excluding those who became institutionalized or had died during each follow-up period: one year (1996–1997, *M* = 29.63, *SD* = 27.68, *n* = 238); two years (1996–1998, *M* = 58.37, *SD* = 45.06, *n* = 227); three years (1996–1999, *M* = 87.21, *SD* = 61.68, *n* = 220); four years (1996–2000, *M* = 116.65, *SD* = 78.20, *n* = 206); and five years (1996–2001, *M* = 149.28, *SD* = 96.83, *n* = 189). Notably, by five years follow-up, the sample size had been reduced by 49 individuals due to deaths and institutionalizations.

## Results

All unidirectional hypotheses were examined with one-tailed tests. Multiple factors contributed to a reduction in sample size from the maximum potential (*n* = 238) for some hypotheses. These included: i) variation in methodology (experimental task vs. survey method), ii) missing values on key variables (denial-of-risk, absence-of-fear, health status), and iii) the use of different follow-up periods that excluded participants who had died or been institutionalized.

### Appraisals Group Differences: Denial-of-Risk and Absence-of-Fear

A priori unidirectional contrasts were tested among respondents who had participated in the experimental task and had full data on key variables (*n* = 64). The mean denial-of-risk scores were contrasted for individuals in the four appraisal groups: invincible (*n* = 10), deficient (*n* = 19), helpless (*n* = 17), and motivated (*n* = 18). As predicted (H1), denial-of-risk was higher for the *invincibles* (*M* = 19.50, *SD* = 25.52) relative to the other groups: deficient [*M* = 6.68, *SD* = 10.19, *t*(60) = -1.84, *p* = .035, Cohen’s *d* = 0.72], helpless [*M* = .88, *SD* = 13.14, *t*(60) = -2.62, *p* = .001, Cohen’s *d =* 0.96], motivated [*M* = 8.06, *SD* = 20.37, *t*(60) = -1.63, *p* = .054, Cohen’s *d =* 0.51]. The strong inclination to deny risk by the *invincibles* was most notable in comparison to the helpless, as reflected by a large effect size using Cohen’s [61] effect size conventions.

Differences across appraisal groups were also found in the analysis of fear that included all individuals who had responded to the fear measure (*n* = 233). As predicted (H2), a significantly higher percentage of the *invincibles* (95%) reported an absence-of-fear relative to those classified as deficient (84%, *χ*^2^ = 2.97, *p* = .043), helpless (79%, *χ*^2^ = 4.57, *p* = .016), or motivated (83%, *χ*^2^ = 3.29, *p* = .035). Thus, only 5% of the *invincibles* reported any fear at all.

These findings show an invincible mindset is characterized by specific cognitions (denial) and emotions (absence-of-fear). To the extent that denial and fearlessness undermine proactive help seeking [42–43], this psychological mindset may be toxic. This speculation provided a platform to consider the detrimental role of the invincible mindset on health care behavior.

### Mindsets and Health Behavior

To examine how psychological mindsets relate to health behavior, the appraisal groups were compared on physician visits over a five-year period (1996–2001). H3 was tested at five follow-up periods. The one-year follow-up analysis was based on the full sample (*n* = 238), whereas, each subsequent follow-up analysis excluded those who had died or been institutionalized. As a result, 49 participants were excluded by the end of the five-year follow-up (*n* = 189). Finally, to test H4 that was also based on the five-year follow-up, one additional individual was excluded due to a missing value on the health status variable (*n* = 188).

### Appraisal Group Differences on Physician Visits

Five separate one-way ANCOVAS were conducted at each follow-up period to test the prediction that the *invincibles* would visit their physicians less frequently than their counterparts (H3), controlling age, gender, income, functional status, and health status. An appraisal group main effect emerged consistently at: one [*F*(3,229) = 3.13, *p* = .026, *n* = 238], two [*F*(3,218) = 4.32, *p* = .006, *n* = 227], three [*F*(3,211) = 4.85, *p* = .003, *n* = 220], four [*F*(3,197) = 3.78, *p* = .011, *n* = 206], and five [*F*(3,180) = 3.15, *p* = .026, *n* = 189] years.

**Fig 2:**
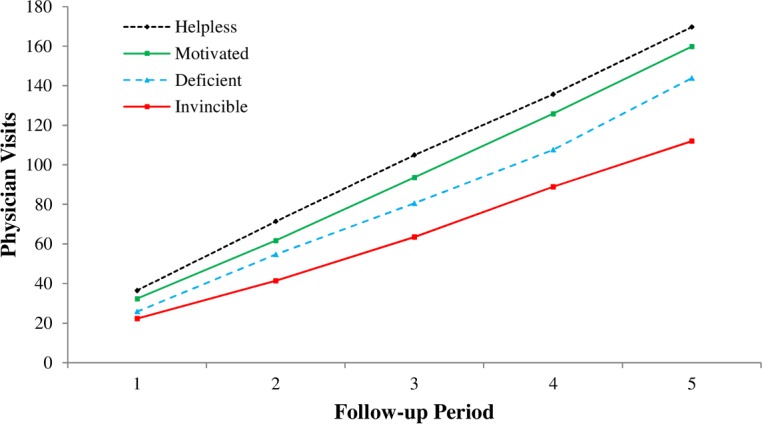
Covariate-Adjusted Mean Number of Physician Visits at Each Follow-Up Period for Appraisal Groups.

Fig 2 presents the (covariate-adjusted) mean physician visits from each of these separate analyses. The important message it depicts is not the overall increase in the number of visits over the years, as would be expected due to the cumulative count of the physician visits over increasing longer follow-up periods. Rather, the primary message is that, at each follow-up, fewer physician visits were made by the *invincibles* relative to other appraisal groups.

Least significance difference tests confirmed H3. By five years, the differences were substantial. Individuals with an invincible mindset made significantly fewer visits (*M* = 111.95) than those in the other groups: deficient [*M* = 143.78, *M diff*(86) = -31.83, *p* = .050, Cohen’s *d* = 0.37], helpless [*M =* 169.67, *M diff*(74) = -57.72, *p* = .003, Cohen’s *d* = 0.67], and motivated [*M =* 159.78, *M diff*(90) = -47.83, *p* = .006, Cohen’s *d* = 0.56]. The differences between those with invincible versus helpless or motivated mindsets conforms to a medium effect size [61].

The appraisal group effects were found after statistically controlling for baseline variables. Although gender and income were non-significant, the other covariates related to physician visits in the expected direction. For example, at five years follow-up, older age [*F*(1,180) = 7.85, *p* < .001], poorer functional status [*F*(1,180) = 5.18, *p* = .024], and poorer health status [*F*(1,180) = 28.46, *p* < .001] were positively related to physician visits. Statistically adjusting for health status indicates that the underutilization (fewer physician visits) by the *invincibles* was not due to them being in better health. Nonetheless, a further analysis directly examined the role of health status in physician visits to consider whether appraisal groups differed in their strategic approach to utilizing health care.

#### Strategic Approaches to Utilizing Health Care

Separate ANOVAs were conducted for each appraisal group to test the health status (good vs. poor health) effect on five-year physician visits (*n* = 188). One-tailed tests were used to consider the underpinning logic that more physician visits should occur when individuals are in poor health because this when it is strategic to seek health care. The predicted effect for health status (good versus poor) was confirmed for three appraisal groups (H4): deficient [*F*(1,53) = 5.65, *p* = .011], helpless [*F*(1,41) = 3.56, *p* = .033], and motivated [*F*(1,57) = 13.59, *p* = .001]. In contrast, the health status effect did not emerge for the *invincibles* [*F*(1,30) = 0.55, *p* = .232], suggesting that poor health did not increase their frequency of physician visits.

**Fig 3:**
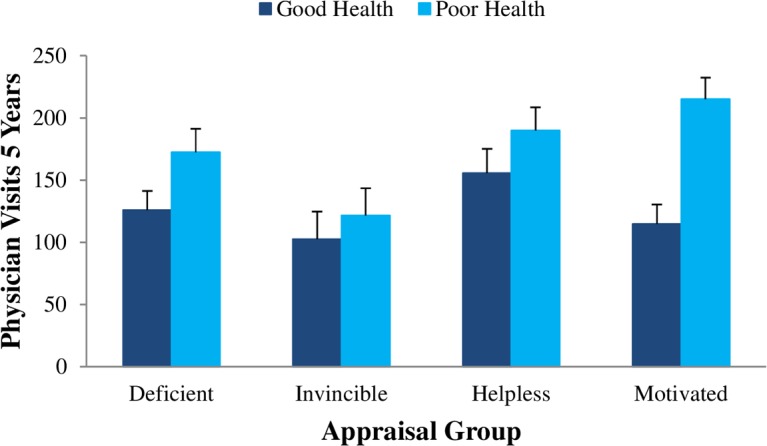
Covariate-Adjusted Mean Number of Physician Visits (Five-Years) for Participants in Poor versus Good Health within Each Appraisal Group.

Fig 3 shows a consistent pattern of higher (covariate-adjusted) mean physician visits for those with poor versus good health status. Notably, however, the very small health status effect was nonsignificant for the *invincibles* (Cohen’s effect size, *d* = 0.23). In contrast, moderate to large effect sizes were found for the helpless (*d* = 0.51), deficient (*d* = 0.54), and motivated (*d* = 1.15) appraisal groups. The large effect for individuals who both valued their health and perceived it as controllable (motivated group) suggests they were the most strategic in their approach to seeking care.

### Alternative Analysis

An alternative analytic approach (OLS regression) was undertaken to test H4. This provided an advantage over the ANOVA approach that tested each appraisal group separately. The regression approach retained a full range of scores on the continuous variables (health status, PC, HV) while directly testing whether appraisals of PC and HV moderated (qualified) the effect of health status on five-year physician visits. Using the PROCESS macro (Model 3) for SPSS, [62] the predicted 3-way Health Status x PC x HV interaction emerged [*F*(1,263) = 3.89, *p* = .049], confirming that PC and HV moderated the effect of health status on physician visits.

The interaction was probed using simple-simple slope analyses to examine the relationship between health status and physician visits at low and high values of PC (low = -1 *SD*, high = +1 *SD*) and HV (low = -1 *SD*, high = +1 *SD*). The results were consistent with the ANOVA findings that showed poor health predicted physician visits for those with deficient, helpless, and motivated mindsets, but not for the *invincibles*. Specifically, when tested at a high level of HV, poor health predicted more physician visits both at low levels of PC [helpless, *b* = .13, *β* = .26, *t*(263) = 1.82, *p* = .035] and at high levels of PC [motivated, *b* = .25, *β* = .50, *t*(263) = 5.44, *p* < .000]. When tested at a low level of HV, poor health again predicted more physician visits at low levels of PC [deficient, *b* = .19, *β* = .37, *t*(263) = 3.28, *p* = .001] but *not* at high levels of PC [*invincibles*, *b* = .07, *β* = .14, *t*(263) = 1.07, *p* = .287]. Thus, an identical conclusion arises from both the regression and ANOVA approaches; being in poor health did not prompt the *invincibles* to visit their physicians.

## Discussion

Despite perceived control (PC) being depicted as a hallmark of successful aging [63], our paradoxical findings suggest that it is detrimental in late life when it contributes to what we describe as an invincible mindset. This highlights the importance of examining PC as part of a broader appraisal system that also includes health value (HV). Our findings show that the invincible mindset (high PC-low HV) is accompanied by other potentially maladaptive cognitive (denial-of-risk) and emotional (absence-of-fear) characteristics and suggest it has detrimental consequences for health behaviors and approaches to seeking care. These findings raise important questions and provide some context for future research.

### An Invincible Mindset

How prevalent is this mindset that seems to involve a misguided sense of invincibility? We identified such a mindset in a small but substantial proportion of adults (17%). Caution in interpreting this finding is needed because another method to classify mindsets could lead to a different result. However, to the extent it is viable to extrapolate to the population of USA adults aged 75+ [64], this would translate into approximately 325,720 (17% of 19,160,000) older Americans who are navigating through life with a sense that they are invincible and with possible consequences for maladaptive health behavior.

The potential that this invincible mindset is toxic is suggested by our findings showing it is also characterized by denial and fearlessness. Denial-of-risk was much higher for the *invincibles*, particularly in relation to their helpless counterparts (*Ms* = 19.5 vs = 0.88), and only 5% of the *invincibles* reported feeling any fear at all. This fearlessness on the part of the *invincibles* is consistent with Pekrun’s control-value theory that proposes emotions such as fear are only activated if an outcome is valued. Perhaps fear is least likely to be triggered when an outcome that is devalued is also appraised as being within one’s control.

To the extent that feeling some fear and recognizing risk facilitates health seeking [42–43], and denial plays a potentially counterproductive role in health promotion behavior [65–66], this may help to account for the under-utilization of heath care among the *invincibles* in our study. They visited their physician less frequently than their counterparts at every point of follow-up, even after controlling for influential covariates (Fig 2). Moreover, the magnitude of the differences was substantial by the end of five years; the *invincibles* visited their physicians 63 fewer times than those with a helpless mindset (raw *Ms* = 110 vs. 173 visits).

Our findings also imply that the *invincible* mindset subverts a strategic approach to seeking care. Although poor health presumably signals a need for care, it did not predict the frequency of physician visits for the *invincibles* (Fig 3). This is in contrast to the pattern of results for individuals in the other appraisal groups who seemed to strategically adjust their health care behavior to match their needs. Whereas the underutilization of health care by the *invincibles* may have few consequences for those who are healthy, significant repercussions could follow for those who are in poor health if diagnoses are missed and treatments are not received. There may also be economic implications for the formal health care system. For example, costs may be initially minimized for the *invincibles* who avoid the health care system, but they would presumably escalate in the long run as health further deteriorates, leading to more hospital admissions and longer stays.

In addition to fostering disengagement from the formal health care system, an invincible mindset might cultivate a more general disengagement from health goals, which could erode quality of day-to-day life and exacerbate daily physical challenges [67]. If an invincible mindset fosters physical dependency, there would be societal implications. The *invincibles* might eventually require more care, increasing burdens for their informal caregivers and creating additional strains on the formal care systems.

Our focus on the maladaptive role of the invincible mindset is not intended to deemphasize the importance of an adaptive combination of appraisals. Individuals with a motivated mindset in which health is valued and perceived as controllable appeared most strategic in their approach. Those in poor (versus good) health had nearly double the number of physician visits (raw *Ms* = 208.2 vs 109.8) by the end of the five-year period. Thus, relative to other groups, the motivated individuals seemed most strategic in adjusting their seeking of care to their level of need (Fig 3). Perhaps this responsiveness to health needs corresponds to a strong capacity to discriminate between when it *is* and *is not* critical to seek care. Although this capacity to discriminate may have been most well developed for motivated individuals, it was not limited to them. Poor health also appeared to cultivate the seeking of care for individuals with deficient and helpless mindsets.

### Strengths and Limitations

Our study had several notable strengths, including access to a representative sample of very old adults that enhances the generalizability of the findings. We also had access to objective physician visits data from a provincial health registry that provided highly reliable indicators of health seeking behavior. A lengthy follow-up period optimized the chance of detecting an impact of psychological appraisals on health seeking behavior. In addition, the design of the SAS study departed from most large-scale correlational studies in that several unique experimental tasks (hypothetical scenarios) were embedded within it [68–69].

In our study, the experimental task that induced threat provided a unique opportunity to assess denial-of-risk. Inducing threat may provide a more sensitive denial-of-risk measure than does a simple self-reported estimate of risk obtained in the absence of threat. Studies in natural settings that attempt to assess risk in the face of threat are difficult since they require recruiting participants who are experiencing real and imminent health threats.

Since threat messages are not taken seriously if they are perceived as unbelievable or irrelevant [70], we carefully designed our task to ensure that the threat was familiar and believable. Nonetheless, our threat induction task could be less sensitive than a real-life threat. To the extent that our measure lacked sensitivity, this would imply that denial-of-risk among the *invincibles’* may be even stronger than what we report in this study.

### Future Directions

Our examination of complexity in psychological mindsets parallels an approach being applied in the study of psychological strategies used to pursue late life health goals. Just as our findings suggest it is maladaptive to simultaneously hold an unlikely combination of appraisals about health (high PC-low HV), certain mindsets consisting of mismatched and conflicting strategies may have negative consequences. For example, we identified a potentially detrimental *unmitigated goal engagement* mindset characterized by strategies that heightened motivation to strive for a goal (e.g., downplaying conflicting goals) in combination with a lack of proactive goal pursuit strategies (e.g., focusing on exerting effort). This mindset that may foster a kind of frustrated procrastination appears maladaptive in that it is associated with low levels of physical activity and poorer cardiorespiratory health over a three-year period [71]. This underscores the importance of examining such counterintuitive combinations of appraisals and strategies.

Future studies should not only examine the maladaptive role of complex mindsets, but should attend to adaptive combinations, such as valuing health and perceiving it as controllable (motivated mindset). In this way, future research can provide new insights for positive psychology that studies how psychological processes work together to promote flourishing. Finally, to the extent that appraisals are modifiable, there is an exciting potential for remedial approaches to shift maladaptive appraisals to more adaptive ways of thinking. Informal interventions could involve physicians simply encouraging their patients to appraise their health in adaptive ways. If maladaptive mindsets can be altered through the physician-patient dialogue, this might offset disengagement and self-neglect. Formal control-enhancing interventions could also be designed using past methods as a platform [72–74]. However, our findings warn against promoting control appraisals if individuals devalue their health. Thus, novel clinical interventions that encourage individuals to appraise their health as controllable should build in adaptive value appraisals, perhaps by encouraging patients to value their health for its own right (intrinsically) and for its role in maintaining functional independence and limiting pain (extrinsically).

### Conclusions

Our findings deepen the knowledge regarding conditions under which PC *is* or *is not* adaptive. Consistent with past findings, results imply that it is adaptive to appraise health as controllable when one also values health [30–31]. This mindset appears to underpin a strategic approach to health care that is likely to foster long-term benefits. In contrast, however, our findings suggest that PC is not a silver bullet. Rather, having a strong perception of control may be counterproductive if it produces a misguided and mistaken sense of invincibility.

Despite our conclusion that an invincible mindset may have detrimental health consequences, the message need not be bleak for such individuals. The malleability of psychological appraisals that underpin this mindset is in stark contrast to the more immutable genetic factors that dictate health. Thus, remedial treatments to undo maladaptive appraisals have the potential to improve the well-being of older adults, ease caregiver burden, and offset costs and mounting pressures facing the health care system.

## Supporting Information

S1 File Dataset containing interview data. (SAV)

S2 File Dataset containing aggregated physician visit data. (SAV)
